# Peripartum Cardiomyopathy Presenting with Predominant Left Ventricular Diastolic Dysfunction: Efficacy of Bromocriptine

**DOI:** 10.1155/2012/476903

**Published:** 2012-11-22

**Authors:** Piercarlo Ballo, Irene Betti, Giuseppe Mangialavori, Leandro Chiodi, Gherardo Rapisardi, Alfredo Zuppiroli

**Affiliations:** ^1^Department of Cardiology, S. Maria Annunziata Hospital, Via dell'Antella 58, 50012 Florence, Italy; ^2^Department of Pediatrics, S. Maria Annunziata Hospital, 50012 Florence, Italy; ^3^Department of Cardiology, Local Health Unit, 50135 Florence, Italy

## Abstract

Management of patients with peripartum cardiomyopathy (PPCM) is still a major clinical problem, as only half of them or slightly more show complete recovery of left ventricular (LV) function despite conventional evidence-based treatment for heart failure. Recent observations suggested that bromocriptine might favor recovery of LV systolic function in patients with PPCM. However, no evidence exists regarding its effect on LV diastolic dysfunction, which is commonly observed in these patients. Tissue Doppler (TD) is an echocardiographic technique that provides unique information on LV diastolic performance. We report the case of a 37-year-old white woman with heart failure (NYHA class II), moderate LV systolic dysfunction (ejection fraction 35%), and severe LV diastolic dysfunction secondary to PPCM, who showed no improvement after 2 weeks of treatment with ramipril, bisoprolol, and furosemide. At 6-week followup after addition of bromocriptine, despite persistence of LV systolic dysfunction, normalization of LV diastolic function was shown by TD, together with improvement in functional status (NYHA I). At 18-month followup, the improvement in LV diastolic function was maintained, and normalization of systolic function was observed. This paper might support the clinical utility of bromocriptine in patients with PPCM by suggesting a potential benefit on LV diastolic dysfunction.

## 1. Introduction

Peripartum cardiomyopathy (PPCM) is a condition of unknown etiology characterized by acute development of heart failure and left ventricular (LV) dysfunction in the last month of pregnancy or within 5 months of delivery [1, 2]. Risk factors include age, multiparity, smoking, African descent, twin pregnancy, tocolytic medications, and pregnancy-related hypertension. The clinical appearance of PPCM resembles that of a dilated cardiomyopathy with acute onset of LV enlargement and dysfunction. Although LV impairment is often reversible, current evidences indicate that nearly 50% of patients show incomplete recovery of LV systolic function after 6–12 months despite conventional evidence-based therapy with ACE-inhibitors, beta-blockers, and diuretics, and that the risk of evolution towards fatal heart failure in this group is not negligible [3–7]. Recently, it has been hypothesized that addition of bromocriptine or other inhibitors of prolactin release to standard therapy may be effective in favoring both clinical status and LV function, and in preventing chronicization of heart failure in subjects with PPCM [8–11]. However, a major issue is that these reports focused only on recovery of LV systolic function—as assessed by echocardiographic measurement of LV ejection fraction—whereas PPCM may often present with predominant LV diastolic dysfunction, or even with advanced diastolic impairment despite normal ejection fraction [12, 13]. To date, the effectiveness of bromocriptine in favoring recovery of LV diastolic function in these patients has never been explored.

**Figure 1:**
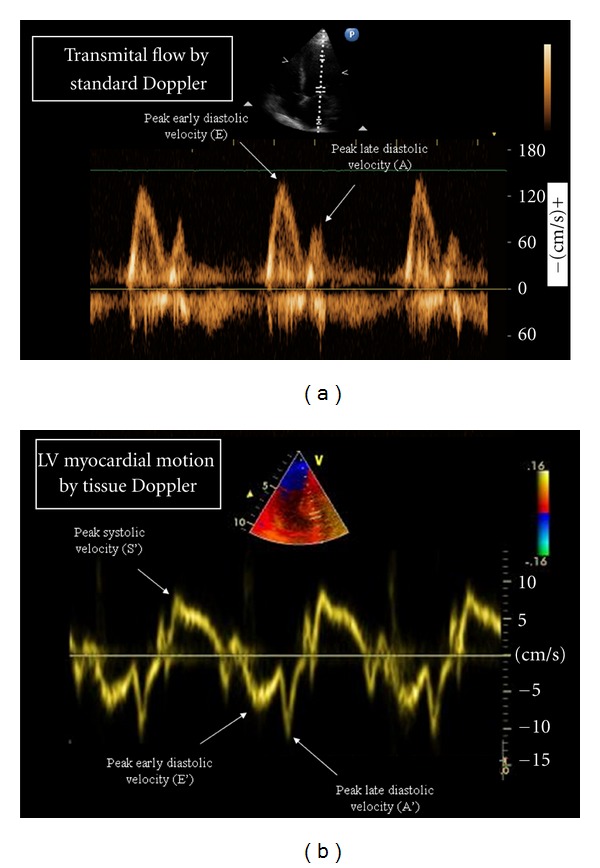
Echocardiographic indexes of left ventricular diastolic function. Standard Doppler of transmitral flow (a) and Tissue Doppler of left ventricular myocardial motion (b). The main indexes for the assessment of left ventricular diastolic function are (1) the peak early diastolic myocardial velocity E′, which is an index of LV relaxation; (2) the ratio between the peak early diastolic velocity of transmitral flow and E′ (E/E′ ratio), which is an index of left ventricular filling pressure.

Echocardiography allows accurate assessment of LV diastolic function. The standard approach was historically based on the assessment of transmitral flow by pulsed Doppler. The typical Doppler pattern of transmitral flow (Figure 1(a)) is characterized by an early diastolic (E) and a late diastolic (A) wave. Although the E/A ratio (normal values >1) has been used for long as an index of diastolic dysfunction, it has important limitations, which include a strict load-dependency and a biphasic course with increasing degrees of diastolic dysfunction, so that patients with advanced diastolic dysfunction often present with an E/A ratio >1 and a Doppler pattern that resembles a normal one (the so-called pseudonormal pattern). Tissue Doppler (TD) imaging of longitudinal mitral annulus motion is an echocardiographic technique that overcomes most of the limitations of the standard assessment based on transmitral flow [14]. The typical TD pattern (Figure 1(b)) is characterized by a systolic positive wave (LV contraction), an early diastolic negative wave (LV relaxation), and a late diastolic negative wave (atrial contraction). The peak early diastolic velocity (E′) is a sensitive and load-independent measure of LV relaxation (normal values >8 cm/s). On the other hand, the ratio between the peak velocity of the E wave of transmitral flow and E′ (normal values <8) is to date considered the reference method for the noninvasive estimation of LV filling pressure, which is the main determinant of symptoms of pulmonary venous congestion in patients with LV diastolic dysfunction [15]. According to the recent ESC guidelines for the Diagnosis and Treatment of Acute and Chronic Heart Failure, an E/E′ ratio of >15 is highly predictive of increased LV filling pressure [16]. An additional application of TD for the assessment of LV diastolic function is provided by the possibility of generating multiple velocity curves in different LV segments, which provide valuable information about intraventricular diastolic dyssynchrony [17].

In this report, we describe the changes in LV diastolic function, as assessed by TD, after 6-week treatment with bromocriptine in a patient with PPCM and severe LV diastolic dysfunction at baseline.

## 2. Case Presentation

**Figure 2:**
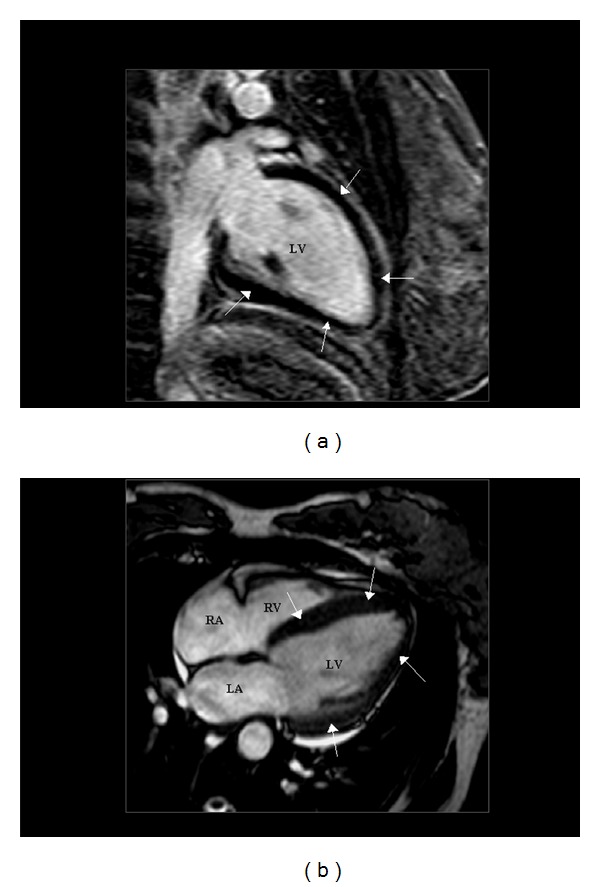
Gadolinium-enhanced cardiac magnetic resonance imaging. Two-chamber (a) and four-chamber (b) views obtained by magnetic resonance imaging after enhancement by gadolinium. In addition to left ventricular enlargement, the dark appearance of the left ventricular myocardium (arrows) indicated lack of delayed enhancement, suggesting that no fibrosis or other abnormalities in myocardial architecture were present. LV = left ventricle; RV = right ventricle; LA = left atrium; RA = right atrium.

A 37-year-old woman with PPCM underwent echocardiographic reevaluation 2 weeks after initial diagnosis. Acute LV enlargement and dysfunction (end-diastolic volume 167 mL, normal values [n.v.] <105 mL; end-systolic volume 109 mL, n.v. <50 mL; ejection fraction 35%, n.v. ≥55%) with clinical evidence of heart failure (NYHA class II) had been diagnosed on day 2 after a normal vaginal delivery. Delivery had occurred at the 36th week of a twin, bicorial, biamniotic pregnancy complicated by a HELLP syndrome. Treatment with ramipril, bisoprolol, and furosemide had been promptly started. Coronary angiography had showed normal coronary arteries, and gadolinum-enhanced cardiac magnetic resonance imaging had confirmed significant LV enlargement (end-diastolic volume 187 mL, end-systolic volume 119 mL) and systolic dysfunction (ejection fraction 36%), with no signs of inflammation or fibrosis, no abnormalities in myocardial perfusion, no signs of right ventricular enlargement or dysfunction, and a mild pericardial effusion (Figure 2). 

**Figure 3:**
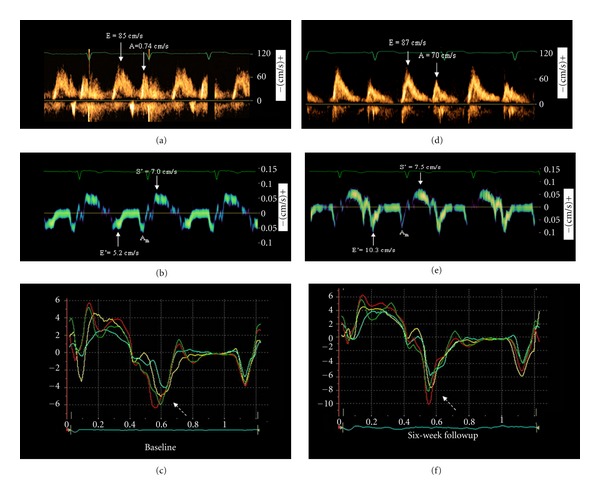
Left ventricular diastolic function at baseline and at the 6-week followup. Left column: Baseline examination. Doppler pattern of transmitral flow (a), Tissue Doppler pattern of left ventricular (LV) motion recorded at the junction with the septal mitral annulus (b), and color Tissue Doppler velocities of multiple LV myocardial segments ((c): yellow, basal septum; turquoise, middle septum; red, basal lateral; green, middle lateral). Severe reduction of peak early diastolic myocardial velocity (E_m_) and considerable early diastolic dyssynchrony between segments (dotted arrow) were present. Right column: corresponding images obtained after 6 weeks of therapy with bromocriptine. Transmitral Doppler pattern was substantially unchanged (d), whereas a considerable increase in E′ (e) and a reduction in early diastolic dyssynchrony ((f), dotted arrow) were observed. Also note the different velocity scales between (c) and (f), and the increase in early diastolic velocities of all myocardial segments at 6-week followup compared to baseline. A = peak late diastolic transmitral flow; A′ = peak late diastolic myocardial velocity; E = peak early diastolic transmitral flow; S′ = peak systolic myocardial velocity.

At the current examination, the patient was still in NYHA class II. Echocardiography showed persistence of LV enlargement and dysfunction (end-diastolic volume 178 mL, end-systolic volume 116 mL, ejection fraction 35%). Doppler imaging of transmitral flow (Figure 3(a)) showed an E/A ratio of 1.1, whereas TD (Figure 3(b)) revealed depressed E′ (septal 5.2 cm/s, lateral 5.6 cm/s), suggesting impaired LV relaxation. The average E/E′ ratio was 15.7, indicating severely increased LV filling pressure. The Valsalva manoeuvre led to an inversion of the E/A ratio, suggesting that the transmitral pattern was pseudonormal. Color TD imaging showed early diastolic dyssynchrony (standard deviation of time-to-peak early diastolic velocities 31.2 ms) (Figure 3(c)). Right ventricular size and function were normal (basal diameter 35 mm, n.v. ≤42 mm; right ventricular outflow tract 22 mm, n.v. ≤26 mm; tricuspid annulus plane systolic excursion 21 mm, n.v. 16 mm; peak systolic tricuspid annulus velocity 14 cm/s, n.v. 10 cm/s) and there were no signs of pulmonary hypertension. A mild, haemodynamically nonsignificant pericardial effusion was still evident, with a maximal end-diastolic dimension of 5 mm at the level of lateral LV wall. Serum NT-proBNP was 215 pg/mL (normal value <125 pg/mL). There were no clinical or electrocardiographic signs of pericarditis, and there was no evidence of infection status or systemic inflammatory disease. C-reactive protein was normal (0.45 mg/dL, n.v. <0.5 mg/dL), and there was no leucocytosis (7.7 *·* 10^3^/*μ*L, n.v. <10.1 *·* 10^3^/*μ*L). Bromocriptine 2.5 mg bid was added to therapy. The patient was discharged, and careful followup was planned.

At 6-week followup, the patient reported improvement in symptoms (NYHA class I). Echocardiography showed an improvement in ejection fraction (45%) with small changes in LV dimensions (end-diastolic volume 175 mL). The mitral E/A ratio was substantially unchanged (1.2), but a considerable increase in E′ (septal 10.3 cm/s, lateral 11.1 cm/s) and a decrease in the E/E′ ratio (8.1) were detected, suggesting improved LV relaxation and decreased LV filling pressure (Figures 3(d) and 3(e)). Accordingly, no inversion of the E/A ratio was observed during the Valsalva maneouvre, suggesting that the transmitral pattern was normal. Color TD time-velocity curves showed a reduction of early diastolic dyssynchrony (standard deviation of time-to-peak early diastolic velocities 10.8 ms) (Figure 3(f)). N-terminal probrain natriuretic peptide was modestly reduced (192 pg/mL). Bromocriptine was well tolerated, and no side effects were observed. Continuation of the treatment and careful followup were recommended. 

At 18-month followup, the patient was still asymptomatic and in good general conditions, and a slight further increase in E′ (septal 10.8 cm/s, lateral 11.8 cm/s) and E/E′ (7.7) was observed, together with normalization of LV ejection fraction (60%). 

## 3. Discussion

Several therapies have been proposed in PPCM in addition to conventional treatment for heart failure, including apheresis, intravenous gamma-globulin, immunomodulators, anti-viral agents, and particularly prolactin release inhibitors such as bromocriptine [18]. The rationale for the use of bromocriptine in PPCM is based on the hypothesis that an increased oxidative stress in the postpartum heart may play a key role in the genesis of PPCM by enhancing the cathepsin D-mediated cleavage of prolactin into its 16-kDa subform, which has angiostatic and proapoptotic properties, promotes vasoconstriction, inhibits endothelial cell proliferation and migration, and favors myocardial microvascular injury [2]. This hypothesis may be supported by the evidence that the failure to decrease prolactin serum concentration is associated with poor outcome in PPCM [19], and by reports showing an improvement in both clinical status and LV ejection fraction after treatment with bromocriptine or other inhibitors of prolactin release [8–10]. In a recent prospective study of African women with newly diagnosed PPCM, in which subjects were randomized to standard care versus standard care plus bromocriptine for 8 weeks, an improvement in LV ejection fraction and a better 6-month outcome was observed in the group treated with bromocriptine as compared to the control group [20]. Unfortunately, the study suffered from some limitations, related to its small sample size (*n* = 20) and to an unexpected excess mortality rate in the control group [21]. Also, these findings may not be applicable to non-African PPCM populations. To date, whether the use of bromocriptine in patients with PPCM could lead to a real prognostic benefit is still debated. A recent study showed that breastfeeding—a major stimulus for the production of prolactin—was significantly associated with recovery of LV systolic function [22]. Cases where bromocriptine did not lead to benefit were also reported [23]. Caution is therefore needed in drawing conclusions about the effectiveness of bromocriptine in patients with PPCM. Moreover, the safety of bromocriptine has been recently questioned, as cases of complications including myocardial infarction and stroke have been observed [23, 24]. 

This report illustrates a case of PPCM with evidence of advanced LV systolic and diastolic impairment, in which an early improvement in TD indexes of LV diastolic function was observed after bromocriptine treatment. Of note, the improvement in measures of LV relaxation and filling pressure was larger than that in systolic ones, so that normalization of LV diastolic function and improvement in NYHA class occurred despite persistence of depressed LV ejection fraction and abnormal N-terminal probrain natriuretic peptide plasma concentration. Interestingly, color TD velocity curves also showed a reduction in early diastolic dyssynchrony at 6-week followup. Considering the initial 2-week persistence of LV dysfunction despite standard therapy, these findings support the hypothesis that bromocriptine might have played a role in determining the observed changes in LV diastolic performance. The association between reduction in mechanical dyssynchrony and improvement in LV diastolic function observed in our patient may also be in accordance with a recent study by Tanaka et al., where resolution of mechanical dyssynchrony was found to be related to a reduction in the E/E′ ratio in a population with acute-onset cardiomyopathy with narrow QRS that included subjects with PPCM [25]. On the other hand, it should be stressed that a direct causal association between bromocriptine treatment and the improvement in LV diastolic function in our patient cannot be demonstrated. In addition, the lack of information about invasively determined LV filling pressure or about pulmonary capillary wedge pressure in our patient represents a limitation for the assessment of LV diastolic dysfunction. Moreover, even if the pericardial effusion observed at baseline was mild and consistent with the small effusions often associated with PPCM, a potential effect on LV diastolic function cannot be excluded. 

Considering the clinical relevance of LV diastolic dysfunction, this report also points out the importance of obtaining adequate information on LV diastole using TD in the serial assessment of patients with PPCM. In accordance with the position statement on PPCM from the Heart Failure Association of the European Society of Cardiology [20], echocardiography should be performed before discharge and after 6 weeks, 6 months, and annually to assess LV function and monitor the efficacy of therapy. Although no mention is made of LV diastolic dysfunction in that document, the important drawbacks related to a definition of PPCM focused only on LV systolic impairment were recently highlighted [12, 13]. In this view, measurement of diastolic indexes by TD during serial echocardiographic evaluations in these patients may be considered a simple, accurate, and inexpensive method to obtain unique information regarding changes in LV diastolic function over time. On the other hand, even if the prognostic value of LV diastolic dysfunction in heart failure is well established, it should also be stressed that the potential prognostic implications of these changes in patients with PPCM remain to be demonstrated. 

In conclusion, this report suggests that (1) bromocriptine might be effective in improving LV diastolic function in PPCM; (2) the improvement can be detected by TD in the early phases of treatment; (3) even if the prognostic impact of LV diastolic dysfunction in the specific population with PPCM is still to be quantified, TD may be considered as a useful tool to assess the impact of therapy on LV diastolic performance in these patients. However, larger randomized, controlled, and possibly blinded comparison studies are needed to provide conclusive evidences about the potential clinical utility of bromocriptine for the treatment of PPCM.
